# Cumulative risk effect of household dysfunction for child maltreatment after intensive intervention of the child protection system in Japan: a longitudinal analysis

**DOI:** 10.1186/s12199-018-0703-6

**Published:** 2018-04-20

**Authors:** Hirotsuna Ohashi, Ichiro Wada, Yui Yamaoka, Ryoko Nakajima-Yamaguchi, Yasukazu Ogai, Nobuaki Morita

**Affiliations:** 1 Department of Social Psychiatry and Mental Health, Faculty of Medicine, Graduate School of Comprehensive Human Sciences, University of Tsukuba, 1-1-1 Tennodai, Tsukuba, Ibaraki 305-8577 Japan; 2 Department of Social Welfare, Hanazono University, 8-1 Nishinokyo Tsubonouchi-cho, Nakagyo-ku, Kyoto, 604-8456 Japan; 3 Aiiku Research Institute, Imperial Gift Foundation Boshi-Aiiku-Kai, 5-6-8 Minami-Azabu, Minato-Ward, Tokyo, Japan; 4 Department of Health Service Research, Faculty of Medicine, University of Tsukuba, 1-1-1 Tennodai, Tsukuba, Ibaraki 305-8577 Japan; 5 Department of Social Psychiatry and Mental Health, Faculty of Medicine, University of Tsukuba, 1-1-1 Tennodai, Tsukuba, Ibaraki 305-8577 Japan

**Keywords:** Household dysfunction, Temporary custody, Child maltreatment recurrence, Intimate partner violence, Substance abuse, Mental health, Multi-type maltreatment, Adverse childhood experience

## Abstract

**Background:**

Building an effective casework system for child maltreatment is a global issue. We estimated the effect of household dysfunction (i.e., interparental violence, caregiver mental health problems, and caregiver substance abuse) on child maltreatment to understand how to advance the current framework of child welfare.

**Methods:**

The sample comprised 759 children (1- to 17-year-old; mean age was 10.6; 404 boys and 355 girls) placed in temporary custody units (one of the strongest intervention of the Japanese child protection system). Caseworkers from 180 units across 43 prefectures completed questionnaires on children and their family and were asked whether a child maltreatment report had been made after cancelation of custody in a 15-month follow-up period. The relations of household dysfunction and maltreatment reports were assessed using the Cox proportional hazard model.

**Results:**

About half (48.4%) of the children had been placed in the unit because of maltreatment, and 88.3% had a history of victimization. Seventy-six cases had maltreatment reports after cancelation. We entered household dysfunction variables individually into the model, and each had a significant relationship with maltreatment reports (hazard ratios for interparental violence, caregiver mental health problem, and substance abuse were 1.69, 1.69, and 2.19, respectively) after covariate adjustment. When treating these three variables as cumulative risk score model of household dysfunction, the hazard ratio increased with increasing number of score (1.96 for score two; 2.35 for score three; score 0 as reference).

**Conclusions:**

Greater household dysfunction score is a risk of maltreatment after intensive intervention. It is imperative to construct systems facilitating cooperation between child and adult service sectors and to deliver seamless services to children and families. Our findings provide child protect services with risk-stratified interventions for children at victimization risk and promote adult-focused services to be proactive in prevention or intervention for adults with perpetration risk.

**Electronic supplementary material:**

The online version of this article (10.1186/s12199-018-0703-6) contains supplementary material, which is available to authorized users.

## Background

The demands placed on the Japanese child protection system (CPS) have been growing over the last decade. Child guidance centers (CGCs), which are the primary agencies for child protection in Japan, received 89,810 reports of maltreatment in 2014—2.6 times the number 10 years prior [1]. It is not uncommon for each social worker to handle more than 100 cases annually. Such an overload has been associated with a substantial number of maltreatment occurrence after CGC involvement, which is an issue of a great public concern. A more sophisticated assessment method is required to prevent adverse incidents.

Numerous studies have identified risk factors for cause of child maltreatment at multiple levels applying ecological-transactional model [2]. At ontogenic level, parent factors include mental health [3] and substance abuse [4, 5]; at microsystemic level, child factors include younger age [3, 6, 7], disability [8], and behavior problems [6, 9, 10] and family factors include being from a single-parent or step-parent family [3, 6, 7], experiencing intimate partner violence (IPV) and conflict [5, 8, 11]; at exosystemic level, community factors include poverty [3, 5, 10] and isolation [8, 12–14]. Additionally, for maltreatment recurrence, the characteristics of the incidence (e.g., types or number of previous episodes of maltreatment) [6, 15], the CPS intervention [3, 6, 11], and post-investigation services [3, 6, 10, 15–17] also contribute.

These risk factors are shown to be interrelated rather than occurring independently [18, 19]. When there are significant interrelations between a group of variables, one way to avoid confounds in estimating their effects is treating them as cumulative variables instead of individual variables. In such strategy, each item is converted into a dichotomous 0 or 1 score and the summed score is entered in analyses [20]. Although explanatory power of each item is lost and equally weighted, we can examine multiple items simultaneously and avoid distorting view of any single item as prominent. Begle et al. estimated parental child maltreatment potential using path analysis based on Belsky’s developmental-ecological model and compared with a model using cumulative risk score [21]. The author concluded that cumulative risk score model was better in fit than path model. In addition, cumulative score does not capitalize sample dependent variables and have generality across studies. The other advantage of this approach is the reduction of the degrees of freedom in multivariate analyses.

There are extensively used cumulative risk score approaches. Averse childhood experience (ACE) is a commonly used measure, which reflects the amount rather than the severity of exposure [22]. When using ACEs, researchers obtain data on experiences of childhood maltreatment (i.e., emotional, physical, and sexual abuse and physical and emotional neglect) and household dysfunction (e.g., caregivers’ substance abuse, mental illness, violent treatment of mother, criminal behavior, parental separation) [23] and use a cumulative stressor model examining the effect of the number of ACEs (ACE score) on outcomes. Studies have indicated that the ACE score has a long-term impact on health and functional outcomes [22–30].

Number of different types of maltreatment (i.e., multi-type maltreatment (MTM) [31]), which is contained in ACEs, is considered a valid measure of the degree of victimization chronicity and is a better predictor of the victim’s status when compared to using only a specific type of maltreatment [31, 32]. It has been demonstrated as a predictor of mental health [33–35] and behavior problems in childhood and adulthood [36–38].

For predictor of child maltreatment, there are some studies using cumulative risk score approach. Wekerle et al. focused relationship between child maltreatment substantiation and six caregiver vulnerability items, such as substance abuse, criminal activities, mental or physical health issue, and lack of social support [39]. They found that total number of caregiver vulnerabilities was better predictor of maltreatment substantiation than any single item. In aforementioned Begle et al.’s study, the authors used 20 items in the risk score for maltreatment potential which included characteristics of parent, child, household, and neighborhood [21]. In the other studies, case type, case duration, and closure result [10] or offender’s history of child maltreatment victimization and the absence of adult watch over the child in community [40] were used in the score that related to child maltreatment recurrence. There are some limitations in these studies for maltreatment predictors. The items included in cumulative score system were different in each study, and meanings of some items are different in culture or social structure. Thus, it is difficult to treat as generalized measure.

In contrast, adapting a commonly used measure such as ACEs is an easy way to generalize estimation across studies. Among ACEs, it is important to focus on household dysfunction (HD), namely intimate partner violence (IPV), substance abuse, and mental illness, because caregivers exhibiting those risks are likely to be involved with medical and social services but are difficult to treat only by the child protective system. Although these factors are important to consider in casework, it can be difficult to use them as integral and weighting clues for delivering appropriate services. Nevertheless, there is little literature focusing on the impact of household dysfunction in child maltreatment casework [39].

### Objective

Our research questions are as follows. First, does household dysfunction predict future child maltreatment occurrence? Relatedly, does MTM influence incidence of maltreatment reports? Although some studies have indicated a relationship between MTM and maltreatment recurrence [41], this relationship remains mostly unclear. Next, does household dysfunction predict future child maltreatment occurrence after adjustment of MTM? We hypothesized that the effect of household dysfunction on maltreatment recurrence would be mediated by current maltreatment status. The results would indicate that failure of casework does not only depend on the maltreatment status but also on the caregiver’s condition. Finally, does the cumulative effect of MTM and household dysfunction contribute to future maltreatment occurrence? This would help in identifying the usefulness of the ACE score in assessing the prognosis of child maltreatment casework.

## Methods

### Design

The current study utilized a longitudinal, non-experimental design. This study was conducted as a part of a nationwide surveillance of temporary custody units in CGCs in 2014, which was in turn part of a child welfare research project.

### Procedure and participants

CGCs are not only specially suited to handling child maltreatment cases but also dealing with the health and behavioral problems of children and caregiver’s adversity. When there is evidence of an urgent need to provide an alternative environment for a child, CGCs will place the child in a temporary custody unit and engage in investigation and treatment of the child and family. After cancelation of custody, the child either receives in-home services after sending back to their home or receives continuous treatment in out-of-home care settings (i.e., institutional or foster parental care). The subjects of the current study were children who had had entered temporary custody between August 1 and 31, 2014. We included only those stayed for more than 3 days, because important information is likely to be missed when children released within few days.

Before starting surveillance, all 207 CGCs in Japan, which spanned 47 prefectures, were contacted to request their participation. The objectives and procedures of the study were explained to the facility heads. One hundred and eighty facilities across 43 prefectures agreed to participate by returning the reply form (participation rate, 86.9%). The caseworkers in the participating facilities completed a questionnaire about the selected children’s cases on three time points, and then, they sent to researchers. The questionnaire assessed various aspects of case records, such as information on the selected child, their parent(s), family environment, history of CGC involvement, and the details of the current casework. Production of questionnaire and data collection were provided by the Aiiku Research Institute (previously the Japan Child and Family Research Institute).

The participants were children who had had entered temporary custody between August 1 and 31, 2014. We included only those who stayed for more than 3 days, because essential information is likely to be missed when children are released within few days. We collected information at three time points: first, about the day of the placement; second, about the day of the cancelation of temporary custody. According to the Japanese Child Welfare Act, “The period for temporary custody … shall not exceed 2 months …” [42]; therefore, when the child’s custody exceeded the 2-month period, we collected data on the 60th day of their placement. This yielded a total of 1081 possible cases. For the third point, the caseworkers completed a follow-up questionnaire on each index child’s condition in January 2015 (about 15 months after the child’s placement). As the government recommends that CGCs should continue providing services for at least 6 months [43], if the casework was terminated before January 2015, the follow-up information reflected their condition at the sixth month after the placement. The information was given for a total of 759 cases (70.2% of 1081 cases).

### Measures

#### Dependent variables

The dependent variable was the existence of maltreatment report after temporary custody cancelation. This was collected from CGC administrative records and coded as present = 1 or not = 0. The date of the report was also obtained.

#### Independent variables

Three types of household dysfunction (IPV, caregiver mental illness, and caregiver substance abuse) and four types of child maltreatment (physical, emotional, and sexual abuse and neglect) were included as independent variables. In Japan, even though children’s exposure to IPV is defined as emotional maltreatment, numerous cases will be closed without sufficient investigation as “children’s problems.” We considered IPV as an aspect of household dysfunction rather than of child maltreatment. Each variable was coded dichotomously. The questions about household dysfunction asked separately for the father and mother were integrated—if neither caregiver had the problem, the case was coded as absent = 0, otherwise coded as present = 1. Japanese national guidelines classify child maltreatment into four categories: physical abuse, emotional abuse, sexual abuse, and neglect [44]. We coded child maltreatment history for each four subtypes dichotomously, present = 1 or absent = 0. Next, we created three cumulative scores: the number of household dysfunction variables (HD score), the number of maltreatment types (MTM score), and a sum of these two cumulative variables.

### Covariates

We selected six covariates from a literature review and the results of a bivariate analysis: age, sex (reference group was male), receipt of public assistance (proxy of economic status), amount of prior CPS involvement, type of post-cancelation treatment (i.e., in-home service or out-of-home service; the former was the reference), and length of stay in temporary custody. We believed that the probability of maltreatment occurrence would be lower if the length of stay was longer, because the observation period of this study was short.

### Statistical analysis

All analyses were performed with R (version 3.2.0 [2015–04–16]; the R Foundation for Statistical Computing). The Cox proportional hazard model was used to investigate the relationship between the independent variables and incidence of maltreatment report after cancelation of temporary custody while controlling for the covariates. To validate the selection of covariates, we conducted a series of partial likelihood ratio and Wald tests according to the purposeful selection method provided by Hosmer et al. [45]. The proportional hazard assumption was evaluated using graphical diagnostics. Overall goodness-of-fit of each model was evaluated by comparing observed and expected numbers of events calculated from martingale residuals in ten groups based on risk score.

### Missing values

Most missing data resulted from CGC workers being unable to obtain sufficient information by the time to return the questionnaire. One hundred eighteen out of the 759 records (15.5%) were incomplete to some degree. Most missing data were for economic status of family (*n* = 37), followed by caregiver divorce (*n* = 17), and family structure (*n* = 7). To prevent data loss from listwise deletion, we filled in missing values using the single imputation method (SIM). To fill in the parent composition item, we coded missing values as a separate category (“unknown”). However, because this method might produce biased results, we employed the multiple imputation (MI) method to further validate the findings [46]. We performed 20 iterations for 30 multiple imputed datasets each. All MI calculations were performed in R using the default settings of the mice 2.25 package [47]. Model parameters were estimated with multivariate Cox regressions applied to each imputed dataset separately. These repeated estimates and their standard errors were combined into final estimates using Rubin’s rule, which ultimately yielded an MI dataset of 759 cases.

## Results

### Descriptive statistics

Table 1 describes the main characteristics of the sample. The mean age of the children at the time of placement was 10.6 years. Half of the children were boys, and 7.5% were non-Japanese. Six out of every ten (58.8%) children were from single-parent families, while around one fifth (20.2%) of children lived with both biological parents. One third (30.8%) of cases were from households receiving public assistance. Regarding reasons for placement, almost half (48.4%) had been placed in temporary custody for protection from maltreatment. The other common reasons were parenting problems (28.6%) and delinquency (14.6%). In total, children with history of any maltreatment accounted for 88.3%; 57.4% of children had a history of physical abuse, 71.3% had emotional abuse, 10.1% had sexual abuse, and 63.2% had neglect. More than one third (37.2%) of children had three types of maltreatment (MTM), in contrast with one eighth (12.8%) were 0—MTM. For household dysfunction, 46.6% of cases had reports of IPV, 43.5% had mental health problems, and 22.5% had substance abuse. Two thirds had some household dysfunction. The number of children with MTM + HD scores 0, 1, 2, 3, 4, 5, and 6 were 49, 79, 134, 197, 151, 116, and 33, respectively.

**Table 1:** Case characteristics

	*n* (%)
Total (*N*)	759
Child characteristics	
Age	
Mean 10.6 (SD 4.0)	
Age group (years)	
1–5	106 (14.0)
6–12	348 (45.8)
13–17	305 (40.2)
Sex	
Male	404 (53.2)
Female	355 (46.8)
Vulnerability	
Behavior problems	386 (50.9)
Family characteristics	
Ethnicity	
Non-Japanese	57 (7.5)
Caregiver divorce	638 (84.1)
Parents	
Biological	153 (20.2)
Step	108 (14.2)
Single	446 (58.8)
Other	45 (5.9)
Unknown	7 (0.9)
Number of siblings	
0	224 (29.5)
1–2	410 (54.0)
3 or more	125 (16.5)
Economic status	
Receiving public assistance	234 (30.8)
CGC case characteristics	
Amount of prior investigations	
0	250 (32.9)
1	151 (19.9)
2–3	183 (24.1)
4 or more	175 (23.1)
Reason for placement	
Child abuse	367 (48.4)
Physical abuse	197 (26.0)
Emotional abuse	116 (15.3)
Sexual abuse	26 (3.4)
Neglect	138 (18.2)
Parenting problem	217 (28.6)
Delinquency	111 (14.6)
Other	64 (8.4)
Post-temporary-custody service	
Out-of-home	239 (31.5)
Household dysfunction	
Interparental violence	354 (46.6)
Mental health problem	330 (43.5)
Substance abuse	171 (22.5)
Child’s history of maltreatment	
Physical abuse	436 (57.4)
Emotional abuse	541 (71.3)
Sexual abuse	77 (10.1)
Neglect	480 (63.2)
Any maltreatment	670 (88.3)
HD score	
0	226 (29.8)
1	274 (36.1)
2	196 (25.8)
3	63 (8.3)
MTM score	
0	97 (12.8)
1	141 (18.6)
2	239 (31.5)
3	282 (37.2)
HD + MTM score	
0	49 (6.5)
1	79 (10.4)
2	134 (17.7)
3	197 (26.0)
4	151 (19.9)
5	116 (15.3)
6	33 (4.3)

*CGC* child guidance center (out-of-home, therapeutic institutions, or foster parents), *HD* household dysfunction (sum of each of the three individual categories of interparental violence, parental substance abuse, and mental health problem), *MTM* multi-type maltreatment (number of types of maltreatment, i.e., physical abuse, emotional abuse, and neglect)

### Cox proportional hazard model

Overall, 76 of the 759 cases had reports of maltreatment within 15-month follow-up period. Bivariate Cox regression models with 30 multiple imputed datasets (Table 2) revealed that the following variables had significant relationships with this outcome: age (pooled hazard ratio [HR], 0.90, 95% confidence interval [CI] [0.85, 0.95]); interparental violence, caregiver mental health problem, and caregiver substance abuse (pooled HR, 1.74, 95% CI [1.10, 2.74]; 1.67, 95% CI [1.06, 2.63]; and 2.13, 95% CI [1.34, 3.40], respectively); physical abuse, emotional abuse, and neglect (pooled HR, 2.71, 95% CI [1.58, 4.65]; 3.08, 95% CI [1.54, 6.17]; and 2.92, 95% CI [1.61, 5.31], respectively); and use of the out-of-home post-temporary custody service (pooled HR, 0.21, 95% CI [0.11, 0.40]). History of sexual abuse and the amount of prior CPS involvement was marginally significantly associated with the outcome (*p* < .25).

**Table 2:** Bivariate Cox proportional hazard regression models for maltreatment report after temporary custody cancelation

	Number	Report	Crude HR	95% CI	*p*
		*n* (%)			
	759	76 (10.0)			
Child characteristics					
Age			0.90	[0.85, 0.95]	< .001
Sex					
Female	355	38 (10.7)	1.15	[0.73, 1.80]	0.554
Behavior problems	386	43 (11.1)	1.30	[0.83, 2.05]	0.250
Family characteristics					
Ethnicity					
Non-Japanese	57	7 (12.3)	1.22	[0.56, 2.65]	0.622
Caregiver divorce	638	71 (11.1)	2.50	[1.01, 6.20]	0.048
Parents					
Biological	153	17 (11.1)	1.00	[ref.]	
Step	108	12 (11.1)	1.03	[0.49, 2.16]	0.934
Single	446	45 (10.1)	0.91	[0.52, 1.59]	0.747
Other	45	1 (2.2)	0.19	[0.03, 1.46]	0.111
Number of siblings			0.97	[0.84, 1.13]	0.740
Receiving public assistance	234	23 (9.8)	0.90	[0.55, 1.47]	0.673
Household dysfunction					
Interparental violence	354	45 (12.7)	1.74	[1.10, 2.74]	0.018
Mental health problem	330	42 (12.7)	1.67	[1.06, 2.63]	0.026
Substance abuse	171	28 (16.4)	2.13	[1.34, 3.40]	0.001
History of maltreatment					
Physical abuse	437	59 (13.5)	2.71	[1.58, 4.65]	< .001
Emotional abuse	545	67 (12.3)	3.08	[1.54, 6.17]	0.002
Sexual abuse	79	4 (5.1)	0.48	[0.17, 1.31]	0.149
Neglect	483	63 (13.0)	2.92	[1.61, 5.31]	< .001
CGC case characteristic					
Amount of prior CGC involvement			0.93	[0.84, 1.02]	0.106
Duration of temporary custody			1.00	[0.99, 1.00]	0.258
Post-temporary custody service					
Out-of-home	239	8 (3.3)	0.21	[0.11, 0.40]	< .001

Note: All analyses were performed with missing values imputed by multiple imputation method

*CGC* child guidance center (out-of-home, therapeutic institutions, or foster parents), *HR* hazard ratio, *CI* confidence interval

Next, we constructed seven Cox regression models, each with one household dysfunction or child maltreatment variable as the independent variable and age, amount of prior CPS involvement before custody, duration of stay in custody, and post-temporary custody service as covariates. Table 3 shows the associations of each independent variable with the outcome, after adjusting for these covariates. Sexual abuse was the only variable without significant relationship with the outcome.

**Table 3:** Multivariate Cox regression for maltreatment report after temporary custody cancelation

	HR	95% CI	*p*
Interparental violence	1.69	[1.06, 2.70]	0.027
Substance abuse	2.19	[1.36, 3.53]	0.001
Mental health problem	1.69	[1.06, 2.72]	0.029
Physical abuse	2.80	[1.61, 4.84]	< .001
Emotional abuse	3.39	[1.67, 6.85]	< .001
Sexual abuse	0.56	[0.20, 1.56]	0.266
Neglect	3.64	[1.99, 6.68]	< .001

Note: All models were adjusted for age, gender, economic status, amount of prior CPS involvement, duration of temporary custody, and post-temporary custody service with missing values imputed by multiple imputation method

*HR* hazard ratio, *CI* confidence interval

Then, we entered the HD and MTM scores (except for sexual abuse, given that no relationship with the outcome; Table 4). The HD score was entered as a four-level (0, 1, 2, and 3) categorical variable with 0 as the reference. For the MTM score, we combined levels 0 and 1 because of the small size; thus, it comprised three levels of 0 to 1, 2, and 3, with 0 to 1 as the reference. For the HD score, the pooled HR had a graded tendency with increasing level (HR was 2.39, 95% CI [1.26, 4.54] for score 2 and 3.17, 95% CI [1.45, 6.92] for score 3). Regarding the MTM score, the HRs for scores 2 and 3 were 2.41, 95% CI [1.09, 5.30] and 6.01, 95% CI [2.91, 12.41], respectively.

**Table 4:** Multivariate Cox regression for maltreatment reports after temporary custody cancelation

	HR	95% CI	Wald test *p*
Household dysfunction score model			
HD score			
0	1.00	[ref.]	
1	1.32	[0.67, 2.62]	0.418
2	2.39	[1.26, 4.54]	0.008
3	3.17	[1.45, 6.92]	0.004
MTM score model			
MTM score			
0–1	1.00	[ref.]	
2	2.41	[1.09, 5.30]	0.029
3	6.01	[2.91, 12.41]	< .001

Note: All models were adjusted for age, amount of prior CPS involvement, duration of temporary custody, and post-temporary custody service with missing values imputed by multiple imputation method

*HR* hazard ratio, *CI* confidence interval, *HD* household dysfunction (sum of each of the three individual categories of interparental violence, parental substance abuse, and mental health problem), *MTM* multi-type maltreatment (number of types of maltreatment, i.e., physical abuse, emotional abuse, and neglect)

Finally, we entered the HD and MTM scores simultaneously (Table 5). When we adjusted for MTM in this model, HD score still significantly predicted the outcome, with the pooled HRs showing a graded tendency (HR for score 2 was 1.96, 95% CI [1.03, 3.73]; HR for score 3 was 2.35, 95% CI [1.07, 5.16]). Then, we added the two scores and entered them as a categorical variable, which was grouped into four levels according to sample size (0 to 1, 2, 3 to 4, and 5 to 6; the reference was 0 to 1). The summed HD score and MTM scores had also a significant relationship with outcome, and its pooled HRs showed a graded tendency with increasing level (HRs for scores of 3 to 4 and 5 to 6 were 5.00, 95% CI [1.53, 16.37] and 11.41, 95% CI [3.47, 37.52], respectively). Among the covariates, age (HR, 0.93, 95% CI [0.88, 0.99]) and a post-temporary custody service of out-of-home service (HR, 0.24, 95% CI [0.13, 0.47]) had significant relationships with the outcome.

**Table 5:** Multivariate Cox regression for maltreatment report after temporary custody cancelation

	HR	95% CI	Wald test *p*
Household dysfunction(HD) and MTM score model			
Covariate			
Age	0.93	[0.87, 0.98]	0.009
Post-temporary custody service			
In-home	1.00	[ref.]	
Out-of-home	0.21	[0.11, 0.39]	< .001
Independent variable			
HD score			
0	1.00	[ref.]	
1	1.22	[0.62, 2.40]	0.571
2	1.96	[1.03, 3.73]	0.041
3	2.35	[1.07, 5.16]	0.034
MTM score			
0–1	1.00	[ref.]	
2	2.12	[0.96, 3.73]	0.064
3	4.82	[2.34, 9.91]	< .001
Composite model			
Covariate			
Age	0.93	[0.88, 0.99]	0.019
Amount of prior CPS involvement	0.95	[0.85, 1.04]	0.27
Duration of temporary custody	1.00	[0.99, 1.00]	0.376
Post-temporary custody service			
In-home	1.00	[ref.]	
Out-of-home	0.24	[0.13, 0.47]	< .001
Independent variable			
HD score + MTM score			
0–1	1.00	[ref.]	
2	2.00	[0.50, 8.01]	0.327
3–4	5.00	[1.53, 16.37]	0.008
5–6	11.41	[3.47, 37.52]	< .001

Note: All analyses were performed with missing values imputed by multiple imputation method

*HR* hazard ratio, *CI* confidence interval, *HD* household dysfunction (sum of each of the three individual categories of interparental violence, parental substance abuse, and mental health problem), *MTM* multi-type maltreatment (number of types of maltreatment, i.e., physical abuse, emotional abuse, and neglect)

### Validation with single imputation method dataset

We then conducted the above analysis using the single imputation method dataset that was coded missing values as a separate category, “unknown”. The HRs of the different levels of the variables exhibited a graded tendency and were larger than the HRs calculated using the SIM. For example, in the household dysfunction model (Additional file 1: Table S1), the HRs for scores of 2 and 3 were 2.31, 95% CI [1.21, 4.40] and 3.25, 95% CI [1.48, 7.12], respectively (in contrast, the HRs in the MI dataset were 2.39 and 3.17; see Table 4). Similarly, for the composite model (sum of the HD score and MTM scores, Additional file 1: Table S2), the HRs for scores 3 to 4 and 5 to 6 were 3.80, 95% CI [1.35, 10.74] and 8.40, 95% CI [2.94, 24.00], respectively (in contrast, the HRs in the MI dataset were 5.00 and 11.41; see Table 5). The above findings indicated that while all of the directions of the coefficients were the same, the size of the effects were larger in the composite model with the MI dataset.

## Discussion

This is the first study to examine maltreatment occurrence of children placed in temporary custody throughout Japan. Based on the description of the sample, children placed in the temporary custody were unique population as they live in adverse condition. Table 1 shows that more than half children were from single-parent families and one third were receiving public assistance. Bivariate model showed (Table 2) that family characteristics such as family structure and economic status were not associated with outcome, i.e., maltreatment report within about 1 year after intensive intervention of temporary custody. Although those family characteristics have been considered as presumable risk factor for child maltreatment in series of precede studies [3, 5–7, 10], the sample bias might make these factors negligible in our analyses. About 90% of children in our study had a history of maltreatment, regardless of their current reason for placement. In addition, most of children whose reason for placement was other than maltreatment had history of child maltreatment (77.5%). The larger the MTM score, the larger the number of samples. These results indicate that chronic victimization [31, 32] pervaded among placed children. Conversely, that may indicate that more victimized children tend to be protected. To substitute MTM + HD score for ACE, the median of number of the adverse experience was three, which is larger than general population. Preceding national survey in other country showed that half of the respondents had no ACEs and those reported four or more ACE were 6.2–8.3% [22, 48]. Summarizing the above, children needed temporary custody is unique population given exposed to multiple adversity.

Regarding seven regression models for each HDs or maltreatment variables (Table 3), adjusted HRs were higher than 1 except for sexual abuse. Some research has shown that sexual abuse was least likely to be re-reported to CPS agencies when compared to physical abuse or neglect [11, 49]. In Japan, sexual abuse is classified as a severe form of maltreatment that requires strong intervention which might make a lower risk of future child maltreatment.

Estimating cumulative effect of adversity (Table 4), both household dysfunction score and MTM score had significant and dose-response relationship with risk of maltreatment report, and the effect of household dysfunction was significant even after adjustment for MTM (Table 5); children with two HDs and three HDs had twofold and 2.5-fold higher risk for maltreatment report after intervention compared to those with no household dysfunction. This finding is comparable to Wekerle et al.’s study [39], where the authors showed relationship between substantiation of maltreatment and number of household dysfunction in cross-sectional analyses. Our study is different in that we conducted longitudinal analyses on children after CPS intervention and estimated the impact of HDs for CPS re-entry for maltreatment after adjustment of the handling difficulty of MTM.

When combining these MTM and HD scores (Table 5), the presence of three types of household dysfunction or maltreatment (out of all six) led to a fourfold higher risk when compared with only one item. One study conducted with young children demonstrated that when a child had more than four ACEs, their mother was more likely to exhibit abusive or neglectful parenting behavior [50]. Our finding partially coincides with this observation. However, we cannot confirm whether sexual abuse has the same relationship, as it was not associated with future maltreatment reports and thus we did not enter it into the cumulative variable analysis.

Among the covariates, younger age was related to a higher risk of a maltreatment report that is compatible with other studies [6, 9, 15, 41]. The other variables, such as economic status, complicated family structure, or family size, are not associated with outcome significantly in bivariate analyses. Although these are indicated as risk factor of child maltreatment occurrence and chronicity in general population, it might be not in the case of children needed advance intervention. Regarding treatment after cancelation of temporary custody, we found that in-home services were associated with a higher risk of a maltreatment report than institutional or foster-parent care. Many studies have identified several important risk factors such as intensity of CPS investigation level, service type, and result of intervention [10, 49, 51]. In Japan, children placed in institutional care are prone to receiving continuous social protection, and thus, they are not likely to be exposed to maltreatment again.

### Implications

Our findings have some implications. First, social workers might use them to provide risk-stratified interventions for maltreatment occurrence or recurrence according to the number of household dysfunction in each case. Moreover, the sum of the number of household dysfunction and maltreatment type and the analogue of ACE may be useful in risk stratification. Next, the child-welfare agency might be more confident in requesting coordination with agencies that provide services for adults. Furthermore, adult-focused services might be more proactive in promoting interventions for adults who exhibit risks of child maltreatment, even if the service is never involved with the index child directly. Because the Japanese CPS is highly burdened already, collaboration with agencies outside this system will be helpful. Focusing on caregiver problems would enable to use resources beyond the framework of the child welfare system. For example, the agencies dealing with mental health problems or substance abuse normally have regular contact with clients and thus can act as gatekeepers for child maltreatment. A Japanese study of 1492 patients with mental health problems including substance abuse found that 9% of patients were living with their children and 38% of them were rearing the child(ren) on their own [52]. For such patients, regardless of the possibility of maltreatment, proactive measures that help them engage in effective parenting and deliver ample social resources are needed. Researches have demonstrated the effectiveness of risk-focused parenting programs, and that parenting characteristics reduce the detrimental effects on children’s mental or behavior outcomes [53]. For IPV exposure, past research showed that resolution of IPV after CPS involvement was significantly associated with the child’s mental and behavior outcomes [54]. Despite that, a lack of coordination between CPS and IPV response agencies is not uncommon [55, 56]. In Japan, because treatment of IPV is beyond the competence of CGC, and workers tend to consider cases of witness IPV as “low risk” or safer than other life-threatening maltreatment incidents, their cases will be closed even without providing sufficient help to children or families.

Additionally, caregivers experiencing such adversity might have other vulnerabilities. Mothers engaging in substance abuse showed that CPS-involved mothers are likely to be younger, have a lower socioeconomic status, and have fewer interpersonal resources compared to those not involved in CPS [57]. Helping IPV victims requires structural interventions, including the contribution of economic, politico-legal, and social environmental factors, and a lack of social support was found to be associated with mental health and parenting functioning [58]. When we focus on these HDs, we should expand the links with other organizations that engage in distinct types of specialist area beyond child-welfare systems and construct multidisciplinary measures aimed at uninterrupted prevention and intervention of child maltreatment in providing effective casework.

### Limitations

A main limitation was the substantial reduction in sample size—namely, around 30% of the original sample was excluded after merging data from the entry and follow-up period. In this study, the information was obtained from separately conducted questionnaire survey in three time points and integrated into longitudinal dataset. Some CGC disagreed to participate at the third time point and that made reduce the sample size. Furthermore, some data had missing values. The small sample size and short observation period allow for limited conclusions. Constructing a comprehensive database for CGC-involved children and their families would allow for more precise analyses in this field. Additionally, because respondents were not actually the index child or family, the variables concerning their experience are likely to be inaccurate, and the severity of victimization was unknown. By obtaining this information in future research, we could provide a more precise guide in constructing effective practice to reduce the severity and chronicity of child maltreatment. In order to implement such research, we should construct self-report system targeted at victimized children and/or their family member other than perpetrators that might be feasible when we use computer-assisted interview that ensure the privacy of participants [59]. Finally, because the study sample was among the most vulnerable population in Japan, one must be cautious in generalizing the findings. Many children in this study were from adverse environment; one third received public assistance and more than half were from single-parent family and suffered from multiple adverse experiences. If we conducted investigation in children without temporary custody placement, the items related to family condition might have strong relationship to outcome, and the group of predictors would be different from this analyses. Despite these limitations, this study is valuable in its demonstration of the importance of number of household dysfunction in predicting the risk of child maltreatment after intensive intervention.

## Conclusion

Our findings suggest that the number of household dysfunctions is a significant predictor of subsequent maltreatment report after intensive CPS intervention. This is true even after adjustment for child maltreatment status and child, family, and service factors. Given the constellation of risks, multidisciplinary system expanded beyond the child welfare system is desired in child maltreatment interventions.

## Additional file

Additional file 1: **Table S1.** Result of analyses using SIM dataset. Multivariate Cox regression for maltreatment reports after temporary custody cancelation. **Table S2.** Result of analyses using SIM dataset. Multivariate Cox regression for maltreatment report after temporary custody cancelation. (DOCX 32 kb)

## Abbreviations

ACE: Adverse childhood experience
CGC: Child guidance center
CPS: Child protection system
HD: Household dysfunction
IPV: Intimate partner violence
MI: Multiple imputation
MTM: Multi-type maltreatment
SIM: Single imputation method
