# Supplementary material for: Cumulative risk effect of household dysfunction for child maltreatment after intensive intervention of the child protection system in Japan: a longitudinal analysis
Source: Environ Health Prev Med. 2018 Apr 20;23:14. doi: 10.1186/s12199-018-0703-6 (PMC5910551; doi:10.1186/s12199-018-0703-6)
Supplement: Supplementary file 1 — Table S1. Result of analyses using SIM dataset. Multivariate Cox regression for maltreatment reports after temporary custody cancelation. Table S2. Result of analyses using SIM dataset. Multivariate Cox regression for maltreatment report after temporary custody cancelation. (DOCX 32 kb) [file 12199_2018_703_MOESM1_ESM.docx]

**Additional file1:**

**Table S1.**

**Result of analyses using SIM dataset.** Multivariate Cox Regression for Maltreatment Reports After Temporary Custody Cancelation

| **Household dysfunction score model** | | | | | | | | | |
| --- | --- | --- | --- | --- | --- | --- | --- | --- | --- |
|  |  | | | | HR | 95% CI | | *z* | Wald test *p* |
| Household dysfunction score | | | | | |  |  |  |  |
|  | | | 0 | | 1 | [ref.] |  |  |  |
|  | | | 1 | | 1.28 | [0.65, | 2.53] | 0.713 | 0.476 |
|  | | | 2 | | 2.31 | [1.21, | 4.40] | 2.544 | 0.011 |
|  | | | 3 | | 3.25 | [1.48, | 7.12] | 2.947 | 0.033 |
| -2 Log L without independent variable | | | | | | | 962.8 |  |  |
| -2 Log L with independent variable | | | | | | | 950.4 |  |  |
| Chi-square of the model (df) | | | | | | | 12.5 (3) |  |  |
| chi-square test *p* | | | | | | | 0.006 |  |  |
|  |  |  | |  | | |  |  |  |
| **MTM score model** | | | | | | | | | |
|  |  | | | | HR | 95% CI | | *z* | Wald test *p* |
| MTM score | | | | | |  |  |  |  |
|  | | | 0–1 | | 1 | [ref.] |  |  |  |
|  | | | 2 | | 2.1 | [1.04, | 4.25] | 2.059 | 0.039 |
|  | | | 3 | | 4.13 | [2.16, | 7.93] | 4.272 | < .001 |
| -2 Log L without independent variable | | | | | | | 962.8 |  |  |
| -2 Log L with independent variable | | | | | | | 940.4 |  |  |
| Chi-square of the model (df) | | | | | | | 22.5 (2) |  |  |
| chi-square test *p* | | | | | | | < .001 |  |  |
| *Note.* All models were adjusted for age, amount of prior CPS involvement, duration of temporary custody, and post-temporary-custody service | | | | | | | | | |
| HR, hazard ratio; CI, confidence interval; -2 Log L, -2 log-likelihood; df, degree of freedom; MTM, multitype maltreatment (number of types of maltreatment) | | | | | | | | | |

**Table S2.**

**Result of analyses using SIM dataset.** Multivariate Cox Regression for Maltreatment Report After Temporary Custody Cancelation

| **Household dysfunction and MTM score model** | | | | | | | | | |
| --- | --- | --- | --- | --- | --- | --- | --- | --- | --- |
|  |  |  | | | HR | 95% CI | | z | Wald test *p* |
| Covariate | | | | |  |  |  |  |  |
|  | Age | | | | 0.91 | [0.86, | 0.97] | -3.122 | 0.002 |
|  | Post-temporary-custody service | | | |  |  |  |  |  |
|  |  | | In-home | | 1 | [ref.] |  |  |  |
|  |  | | Out-of-home | | 0.21 | [0.10, | 0.45] | -4.096 | < .001 |
| Independent variable | | | | |  |  |  |  |  |
|  | Household dysfunction score | | | |  |  |  |  |  |
|  |  | | 0 | | 1 | [ref.] |  |  |  |
|  |  | | 1 | | 1.18 | [0.60, | 2.32] | 0.472 | 0.637 |
|  |  | | 2 | | 1.99 | [1.04, | 3.79] | 2.085 | 0.037 |
|  |  | | 3 | | 2.53 | [1.15, | 5.57] | 2.297 | 0.022 |
|  | MTM score | | | |  |  |  |  |  |
|  |  | | 0–1 | | 1 | [ref.] |  |  |  |
|  |  | | 2 | | 1.85 | [0.91, | 3.75] | 1.692 | 0.091 |
|  |  | | 3 | | 3.36 | [1.75, | 6.44] | 3.655 | < .001 |
|  | -2 Log L without independent variable | | | | |  | 964.1 |  |  |
|  | -2 Log L with independent variable | | | | |  | 935.5 |  |  |
|  | Chi-square of the model (df) | | | | |  | 28.7 (5) |  |  |
|  | Chi-square test *p* | | | | |  | < .001 |  |  |
|  |  |  | | |  |  |  |  |  |
| **Composite model** | | | | | | | | | |
|  |  |  | | | HR | 95% CI | | z | Wald Test *p* |
| Covariate | | | | |  |  |  |  |  |
|  | Age | | | | 0.92 | [0.87, | 0.98] | -2.73 | 0.006 |
|  | Amount of prior CPS involvement | | | | 0.93 | [0.84, | 1.02] | -1.486 | 0.137 |
|  | Duration of temporary custody | | | | 1 | [0.99, | 1.01] | -0.832 | 0.405 |
|  | Post-temporary custody service | | | |  |  |  |  |  |
|  |  | | | In-home | 1 | [ref.] |  |  |  |
|  |  | | | Out-of-home | 0.25 | [0.12, | 0.52] | -3.643 | < .001 |
| Independent variable | | | | |  |  |  |  |  |
|  | Household dysfunction | | | |  |  |  |  |  |
|  | + MTM score | | | |  |  |  |  |  |
|  |  | | | 0–1 | 1 | [ref.] |  |  |  |
|  |  | | | 2 | 1.49 | [0.42, | 5.27] | 0.613 | 0.54 |
|  |  | | | 3–4 | 3.80 | [1.35, | 10.74] | 2.519 | 0.012 |
|  |  | | | 5–6 | 8.40 | [2.94, | 24.00] | 3.971 | < .001 |
|  | -2 Log L without independent variable | | | | |  | 962.8 |  |  |
|  | -2 Log L with independent variable | | | | |  | 930.3 |  |  |
|  | Chi-square of the model (df) | | | | |  | 32.59 (3) |  |  |
|  | Chi-square test *p* | | | | |  | < .001 |  |  |
| *Note.* HR, hazard ratio; CI, confidence interval; -2 Log L, -2 log-likelihood; df, degree of freedom | | | | | | | | | |
